# Minimally Invasive Coronary Artery Bypass Grafting in Patients With Prior Myocardial Infarction: A Propensity Score-Matched Retrospective Study

**DOI:** 10.31083/RCM49786

**Published:** 2026-09-16

**Authors:** Yanbin Lei, Guang Li, Zhao Yang, Bin You

**Affiliations:** ^1^Department of Cardiovascular Surgery, Minimally Invasive Cardiac Surgery Center, Beijing Anzhen Hospital, Capital Medical University, 100029 Beijing, China

**Keywords:** coronary artery bypass grafting, minimally invasive surgery, myocardial infarction

## Abstract

**Background:**

Patients with prior myocardial infarction (MI) represent a high-risk subgroup for coronary artery bypass grafting (CABG). The primary goal of CABG is durable myocardial revascularization to prevent recurrent ischemic events and improve survival. Minimally invasive CABG (MICS-CABG) may accelerate recovery, but evidence specifically in patients with a history of MI remains limited.

**Methods:**

We performed a single-center retrospective cohort study of adults with prior MI who underwent isolated CABG from 2017 to 2024. Patients with new or recurrent MI within 3 months were excluded. Patients underwent either MICS-CABG via left mini-thoracotomy or conventional CABG via median sternotomy. Propensity score matching (PSM; 1:1) was used to balance preoperative covariates. The primary outcome was the occurrence of major adverse cardiovascular events (MACEs) during the index hospitalization, reflecting the safety and adequacy of surgical myocardial revascularization. Secondary outcomes included postoperative length of stay (LOS), intensive care unit stay, 24-hour drainage volume, and in-hospital mortality. Univariable and multivariable linear regression analyses were performed to evaluate factors associated with postoperative LOS.

**Results:**

The unmatched cohort included 208 patients (sternotomy CABG, n = 142; MICS-CABG, n = 66). After PSM, 106 patients (53 pairs) were included in the analysis. The number of grafts was lower in the MICS-CABG group both before matching (1.71 ± 0.84 vs. 2.08 ± 0.91; *p* = 0.004) and after matching (1.79 ± 0.86 vs. 2.17 ± 0.89; *p* = 0.029). The number of diseased vessels was also lower in the MICS-CABG group after matching (1.83 ± 0.85 vs. 2.17 ± 0.83; *p* = 0.039). The incidence of in-hospital MACEs was low and did not differ between groups, and no MACEs occurred after matching. Postoperative LOS was shorter with the minimally invasive approach before matching (6.06 ± 2.79 vs. 7.20 ± 2.98 days; mean difference, −1.14 days; 95% confidence interval (CI), −1.97 to −0.31; *p* = 0.009) and after matching (6.09 ± 2.96 vs. 7.34 ± 2.68 days; mean difference, −1.25 days; 95% CI, −2.32 to −0.18; *p* = 0.025). However, this association was attenuated and no longer significant after multivariable adjustment. Early postoperative complications, support therapies, and in-hospital mortality were uncommon and comparable between groups, and no reoperations occurred.

**Conclusions:**

In selected patients with prior MI, MICS-CABG demonstrated comparable early safety and a similar incidence of in-hospital MACEs compared with conventional sternotomy CABG after PSM. Although the diseased/transplanted vessel ratio was similar between groups, this finding should be interpreted cautiously because the metric does not fully capture angiographic complexity or graftability. Although the minimally invasive approach was associated with a shorter postoperative LOS in unadjusted analyses, this association was attenuated and not statistically significant after multivariable adjustment.

## 1. Introduction

Coronary artery bypass grafting (CABG) remains the reference revascularization strategy for patients with complex multivessel coronary artery disease (CAD), left main disease, and ischemic cardiomyopathy, for whom contemporary guidelines and randomized evidence support the increased durability of surgical revascularization compared with percutaneous coronary intervention (PCI) [1,2,3,4,5,6,7]. The primary therapeutic goal of CABG is to achieve effective and durable myocardial revascularization, thereby reducing recurrent ischemic events and improving long-term survival in patients with advanced coronary disease. Patients with prior myocardial infarction (MI) represent a clinically important subgroup because infarct-related ventricular remodeling, myocardial scar, residual ischemia, and diffuse coronary atherosclerosis can increase operative complexity and perioperative vulnerability [8,9].

Minimally invasive CABG (MICS-CABG), most commonly performed through a left mini-thoracotomy, has emerged as an alternative to conventional median sternotomy in selected patients. Thus, by avoiding sternotomy and minimizing chest wall disruption, MICS-CABG may facilitate earlier recovery, better pain control, improved mobilization, enhanced pulmonary mechanics, and earlier readiness for discharge, while preserving the fundamental objective of surgical revascularization [10,11,12]. However, in coronary surgery, minimally invasive access is a technical approach rather than the primary therapeutic goal; any potential recovery-related benefits, including shorter hospital stay, should be considered secondary outcomes and should not compromise the completeness or durability of myocardial revascularization.

Contemporary observational and multi-institutional studies suggest acceptable early outcomes and resource utilization in carefully selected patients, and long-term data from minimally invasive direct CABG (MIDCAB) programs support procedural durability in experienced centers. However, evidence focused on patients with prior MI remains limited because many reports include mixed-risk cohorts or do not stratify outcomes by infarct history [13,14]. Therefore, concerns persist regarding hemodynamic tolerance to cardiac manipulation, the feasibility of complete revascularization through limited access, and the potential need for perioperative mechanical circulatory or organ support.

Accordingly, we performed a propensity score-matched retrospective study to evaluate the safety and perioperative efficacy of MICS-CABG in patients with prior MI at a high-volume CABG center. We compared baseline characteristics, operative data, and early postoperative outcomes, including postoperative length of stay (LOS) and a composite of major adverse cardiovascular events (MACEs), between patients with prior MI who underwent MICS-CABG and those who underwent conventional sternotomy CABG. We hypothesized that, in carefully selected patients with prior MI, MICS-CABG could achieve comparable short-term safety and revascularization adequacy while offering potential recovery advantages, such as shorter postoperative hospitalization.

## 2. Methods

### 2.1 Study Design and Patient Population

This single-center, retrospective cohort study was conducted at a high-volume tertiary cardiac surgery center. The institutional CABG database was queried to identify all adult patients (≥18 years) who underwent isolated CABG between January 2017 and December 2024. The present analysis included only patients with a documented history of MI and excluded those who underwent concomitant valve or aortic procedures. Patients with missing data for variables required for propensity score estimation or missing primary outcome data were also excluded. After applying these criteria, 208 patients with prior MI were included and categorized according to surgical approach: conventional median sternotomy CABG (sternotomy CABG; n = 142) and MICS-CABG via a left mini-thoracotomy (n = 66). All operations were performed as isolated CABG procedures.

The study was conducted in accordance with the Declaration of Helsinki. The study was approved by the Ethics Committee of Beijing Anzhen Hospital (Ethics No: Approval No. 2026006x), and the requirement for informed consent was waived because of the retrospective study design.

### 2.2 Surgical Technique

In the sternotomy group, a standard median sternotomy was performed under general anesthesia. Internal thoracic artery and saphenous vein grafts were used at the discretion of the surgeon, based on coronary anatomy and patient characteristics. Off-pump CABG was preferred, whereas cardiopulmonary bypass (CPB) with aortic cross-clamping was used selectively based on intraoperative findings.

In the MICS-CABG group, patients were positioned supine with slight elevation of the left hemithorax. A left anterolateral mini-thoracotomy was performed in the fourth or fifth intercostal space for conduit harvest and distal anastomoses on the beating heart. One-lung ventilation was used when necessary to optimize exposure. The choice between off-pump and on-pump strategies, as well as the number and configuration of grafts, was determined by the operating surgeon. Proximal anastomoses were constructed on the ascending aorta under partial clamping or with dedicated aortic devices, depending on aortic calcification and technical considerations. Perioperative anesthetic, hemodynamic, and intensive care management followed institutional protocols and were broadly similar between the two approaches. The operative goal in both surgical approaches was to achieve complete myocardial revascularization whenever technically feasible. The final number and configuration of grafts were determined based on coronary anatomy, target-vessel quality, and intraoperative technical considerations.

### 2.3 Definition of Prior MI and Exclusion of Recent MI

Prior MI was defined based on a documented clinical diagnosis and objective evidence in the medical record, consistent with the Fourth Universal Definition of MI. Specifically, prior MI was considered present if there was a prior physician-documented diagnosis of MI and/or evidence of MI supported by myocardial injury biomarkers with compatible electrocardiographic findings, and/or imaging evidence of infarction/scar consistent with an ischemic etiology on echocardiography. Biomarker and electrocardiographic evidence referred to the index infarction event documented in prior medical records rather than to perioperative measurements. Patients with new or recurrent MI within 3 months before surgery were excluded.

### 2.4 Variables and Outcomes

Preoperative variables included age, sex, body mass index, hypertension, diabetes, smoking status, hyperlipidemia, prior stroke, prior PCI, renal function (estimated glomerular filtration rate), left ventricular ejection fraction (LVEF), EuroSCORE II, and the grades of mitral, aortic, and tricuspid regurgitation. The exposure was a surgical approach (MICS-CABG vs. sternotomy CABG), as documented in the operative report.

Intraoperative variables included operative time (hours), CPB use (yes/no), packed red blood cell transfusion volume (units), and number of grafts. Early postoperative variables included 24-hour drainage volume (mL), intensive care unit (ICU) stay (hours), and postoperative LOS (days), defined as the time from the index operation to hospital discharge. The diseased/transplanted vessel ratio was defined as the number of diseased major coronary arteries (≥70% stenosis or ≥50% left main stenosis) divided by the number of grafts placed.

The primary outcome was the occurrence of MACEs during the index hospitalization, reflecting the safety and adequacy of surgical myocardial revascularization. Secondary outcomes included postoperative LOS, ICU stay, 24-hour drainage volume, and in-hospital mortality. Univariable and multivariable linear regression analyses were performed to evaluate factors associated with postoperative LOS.

MACEs were defined as a composite of stroke, MI, repeat revascularization (PCI or surgical reintervention), all-cause in-hospital death, clinically significant arrhythmia, and use of advanced support therapies (intra-aortic balloon pump (IABP), extracorporeal membrane oxygenation (ECMO), or continuous renal replacement therapy (CRRT)). Clinically significant arrhythmia was defined as sustained ventricular tachycardia/ventricular fibrillation or any rhythm disturbance requiring electrical cardioversion/defibrillation, temporary pacing, or intravenous antiarrhythmic therapy.

### 2.5 Statistical Analysis

Continuous variables were evaluated for distributional characteristics and are presented as mean alongside the standard deviation (SD) for approximately normal distributions or median (interquartile range) for skewed distributions, as appropriate. Categorical variables are reported as counts and percentages. Between-group comparisons were performed using the Student’s *t* test or Mann–Whitney U test for continuous variables and the χ^2^ test or Fisher’s exact test for categorical variables, as appropriate. All tests were two-sided, and *p* < 0.05 was considered statistically significant.

To reduce baseline confounding, propensity score matching (PSM) was performed to create comparable cohorts undergoing MICS-CABG or sternotomy CABG. Propensity scores were estimated using logistic regression with surgical approach as the dependent variable and prespecified preoperative covariates (age, sex, body mass index, hypertension, diabetes, smoking status, hyperlipidemia, history of stroke, history of PCI, estimated glomerular filtration rate, LVEF, EuroSCORE II, and valvular regurgitation grades). Patients were matched 1:1 using nearest-neighbor matching without replacement, with a caliper of 0.2 standard deviations of the logit of the propensity score. Covariate balance was assessed using standardized mean differences (SMDs), with an absolute SMD <0.10 indicating adequate balance, and visualized using a Love plot.

Postoperative LOS was analyzed as a continuous outcome. Univariable and multivariable linear regression models were fitted to identify factors associated with LOS, with regression coefficients (β) and 95% confidence intervals (CIs) reported. The multivariable model included surgical approach (MICS vs. sternotomy) and prespecified covariates (age, diabetes, EuroSCORE II, LVEF, CPB use, and number of grafts). To assess the robustness of the findings, a sensitivity analysis was performed, excluding postoperative variables (CPB use and number of grafts) from the multivariable model.

Prespecified subgroup analyses evaluated the consistency of the association between surgical approach and postoperative LOS across categories of age, sex, BMI, diabetes status, hypertension, hyperlipidemia, and LVEF, summarized as mean differences with 95% confidence intervals and displayed in a forest plot. Learning effects were explored by plotting operative time against case sequence and by cumulative sum (CUSUM) analysis to identify potential inflection points. No multiple imputation was performed. Patients with substantial missing data in key variables required for matching or modeling were excluded; the remaining analyses were conducted as complete-case analyses. All analyses were performed in R (version 4.3.2; R Foundation for Statistical Computing, Vienna, Austria) using MatchIt for PSM, cobalt for balance diagnostics, and ggplot2 for figures.

## 3. Results

A total of 208 patients were included in the unmatched cohort (sternotomy CABG, n = 142; MICS-CABG, n = 66). After 1:1 PSM, 106 patients (53 matched pairs) were retained for the matched analyses. Baseline characteristics and perioperative outcomes are summarized in Table 1. In the unmatched cohort, most preoperative variables were similar between groups; however, patients in the MICS-CABG group more frequently had a history of PCI (42.4% vs. 16.9%; *p* < 0.001) and had a slightly higher LVEF (57.77 [8.97] vs. 54.71 [9.96]; *p* = 0.035). After matching, between-group differences in measured preoperative characteristics were largely attenuated (Table 1).

**Table 1.** **Baseline characteristics and perioperative outcomes before and after propensity score matching (PSM)**.

Characteristic		Pre-PSM (Sternotomy N = 142)	Pre-PSM (MICS N = 66)	*p*	After-PSM (Sternotomy N = 53)	After-PSM (MICS N = 53)	*p*
Preoperative baseline							
Age, years		62.80 (8.72)	60.89 (9.81)	0.161	61.68 (10.18)	61.85 (9.76)	0.930
Sex, male (%)		99 (69.7)	54 (81.8)	0.094	42 (79.2)	42 (79.2)	1.000
BMI (kg/m^2^)		25.66 (3.09)	25.31 (3.19)	0.445	25.89 (3.17)	25.42 (3.30)	0.460
Hypertension (%)		74 (52.1)	33 (50.0)	0.893	27 (50.9)	27 (50.9)	1.000
Diabetes (%)		73 (51.4)	24 (36.4)	0.061	21 (39.6)	22 (41.5)	1.000
Smoking (%)		77 (54.2)	45 (68.2)	0.080	33 (62.3)	34 (64.2)	1.000
Hyperlipidemia (%)		43 (30.3)	15 (22.7)	0.335	12 (22.6)	12 (22.6)	1.000
History of stroke (%)		22 (15.5)	14 (21.2)	0.413	10 (18.9)	9 (17.0)	1.000
History of PCI (%)		24 (16.9)	28 (42.4)	<0.001	17 (32.1)	15 (28.3)	0.832
eGFR, mL/min/1.73 m^2^		92.5 (84.0, 99.0)	93.5 (83.0, 95.0)	0.520	93.0 (84.8, 99.5)	93.0 (82.7, 93.2)	0.405
LVEF (%)		54.71 (9.96)	57.77 (8.97)	0.035	56.49 (8.65)	57.01 (9.52)	0.769
EuroSCORE II		1.52 (0.88)	1.32 (0.57)	0.099	1.32 (0.54)	1.33 (0.51)	0.903
MR grade		0.90 (0.67)	0.73 (0.54)	0.065	0.79 (0.69)	0.75 (0.55)	0.756
AR grade		0.33 (0.47)	0.33 (0.48)	0.973	0.26 (0.45)	0.28 (0.45)	0.830
TR grade		0.38 (0.52)	0.41 (0.53)	0.710	0.40 (0.53)	0.38 (0.53)	0.855
Intraoperative variables							
Operation time (h)		4.29 (0.85)	4.23 (1.00)	0.689	4.38 (1.14)	4.25 (1.06)	0.539
CPB use (%)		6 (4.2)	1 (1.5)	0.551	2 (3.8)	1 (1.9)	1.000
RBC transfusion (units)		0.35 (1.26)	0.33 (1.43)	0.952	0.55 (1.76)	0.42 (1.59)	0.793
Number of diseased vessels		2.11 (0.87)	1.80 (0.85)	0.016	2.17 (0.83)	1.83 (0.85)	0.039
Number of grafts		2.08 (0.91)	1.71 (0.84)	0.004	2.17 (0.89)	1.79 (0.86)	0.029
Grafts category (%)				<0.001			0.052
	1 graft	35 (24.6)	30 (45.5)		14 (26.4)	26 (49.1)	
	2 grafts	58 (40.8)	26 (39.4)		19 (35.8)	12 (22.6)	
	≥3 grafts	49 (34.5)	10 (15.2)		20 (37.7)	15 (28.3)	
Diseased/transplanted vessel ratio		1.0 (1.0, 1.0)	1.0 (1.0, 1.0)	0.750	1.0 (1.0, 1.0)	1.0 (1.0, 1.0)	0.834
Early postoperative outcomes							
24-hour drainage volume (mL)		386.69 (214.13)	400.12 (219.02)	0.676	420.42 (211.76)	400.00 (205.43)	0.615
ICU stay (h)		48.5 (44.5, 52.0)	46.5 (43.0, 49.0)	0.042	48.1 (44.2, 50.2)	46.7 (46.0, 47.6)	0.323
Postoperative length of stay (d)		7.20 (2.98)	6.06 (2.79)	0.009	7.34 (2.68)	6.09 (2.96)	0.025
Reoperation rate (%)		0 (0.0)	0 (0.0)	NA	0 (0.0)	0 (0.0)	NA
In-hospital mortality (%)		3 (2.1)	0 (0.0)	0.572	1 (1.9)	0 (0.0)	1.000
MACEs		9 (6.3)	3 (4.5)	0.756	0 (0.0)	0 (0.0)	NA
IABP		7 (4.9)	2 (3.0)	0.722	2 (3.8)	2 (3.8)	1.000
ECMO		1 (0.7)	0 (0.0)	1.000	1 (1.9)	0 (0.0)	1.000
CRRT		2 (1.4)	1 (1.5)	1.000	1 (1.9)	1 (1.9)	1.000

Variables with skewed distributions, including eGFR and ICU stay, are presented as the median (Q1–Q3). Other continuous variables are presented as mean (SD). The *p*-values were calculated using the Student’s *t* test for normally distributed continuous variables and the Mann–Whitney U test for non-normally distributed continuous variables. Categorical variables were compared using the χ^2^ test or Fisher’s exact test, as appropriate. The *p*-values were not calculated when no events occurred in either group. RBC transfusion is reported as units of packed red blood cells. Abbreviations: AR, aortic regurgitation; CPB, cardiopulmonary bypass; CRRT, continuous renal replacement therapy; ECMO, extracorporeal membrane oxygenation; eGFR, estimated glomerular filtration rate; LVEF, left ventricular ejection fraction; IABP, intra-aortic balloon pump; ICU, intensive care unit; MACEs, major adverse cardiovascular events; MICS, label for the MICS-CABG group; MR, mitral regurgitation; PCI, percutaneous coronary intervention; TR, tricuspid regurgitation; PSM, propensity score matching; EuroSCORE II, European System for Cardiac Operative Risk Evaluation II.

Intraoperative outcomes are shown in Table 1. Operative time did not differ significantly between groups in either the unmatched (4.29 [0.85] vs. 4.23 [1.00] h; *p* = 0.689) or matched cohorts (4.38 [1.14] vs. 4.25 [1.06] h; *p* = 0.539). CPB was infrequently used and was comparable between groups (unmatched: 4.2% vs. 1.5%; *p* = 0.551; matched: 3.8% vs. 1.9%; *p* = 1.000). RBC transfusion requirements were also comparable (unmatched: 0.35 [1.26] vs. 0.33 [1.43] units; *p* = 0.952; matched: 0.55 [1.76] vs. 0.42 [1.59] units; *p* = 0.793). The number of diseased vessels was lower in the MICS-CABG group both before and after matching (Table 1). To further describe revascularization in relation to coronary disease burden, we calculated the diseased-to-transplanted vessel ratio. The median ratio was 1.0 (IQR 1.0–1.0) in both groups, with no significant difference between the sternotomy and MICS groups (*p* = 0.834). However, this ratio is an exploratory metric and does not fully capture angiographic complexity, target-vessel quality, or graftability. The number of grafts was lower in the MICS-CABG group in both the unmatched cohort (1.71 [0.84] vs. 2.08 [0.91]; *p* = 0.004) and the matched cohort (1.79 [0.86] vs. 2.17 [0.89]; *p* = 0.029). To improve the characterization of operative strategy, the number of grafts was further categorized as 1, 2, or ≥3. The distribution of graft numbers did not differ significantly between the two groups in the matched cohort (*p* = 0.052). However, a higher proportion of patients in the sternotomy group received ≥3 grafts.

Early postoperative outcomes are presented in Table 1. Twenty-four-hour drainage volume was comparable between groups in both cohorts. ICU stay was slightly shorter in the MICS-CABG group before matching (*p* = 0.042), but the difference was no longer significant after PSM (*p* = 0.323). Postoperative LOS was shorter in the MICS-CABG group in both the unmatched cohort (6.06 [2.79] vs. 7.20 [2.98] days; mean difference, −1.14 days; 95% CI, −1.97 to −0.31; *p* = 0.009) and after matching (6.09 [2.96] vs. 7.34 [2.68] days; mean difference, −1.25 days; 95% CI, −2.32 to −0.18; *p* = 0.025). The distribution of LOS by approach is shown in Fig. 1. No reoperations occurred in either group. In-hospital mortality was low and did not differ significantly (unmatched: 2.1% vs. 0.0%; *p* = 0.572; matched: 1.9% vs. 0.0%; *p* = 1.000). In the unmatched cohort, MACEs were comparable between groups (6.3% vs. 4.5%; *p* = 0.756); after matching, no MACEs were observed in either group. Use of IABP, ECMO, and CRRT did not differ significantly between groups in either cohort (all *p* > 0.05). Event rates were low, and several matched outcomes had no events in either group.

**Fig. 1.**
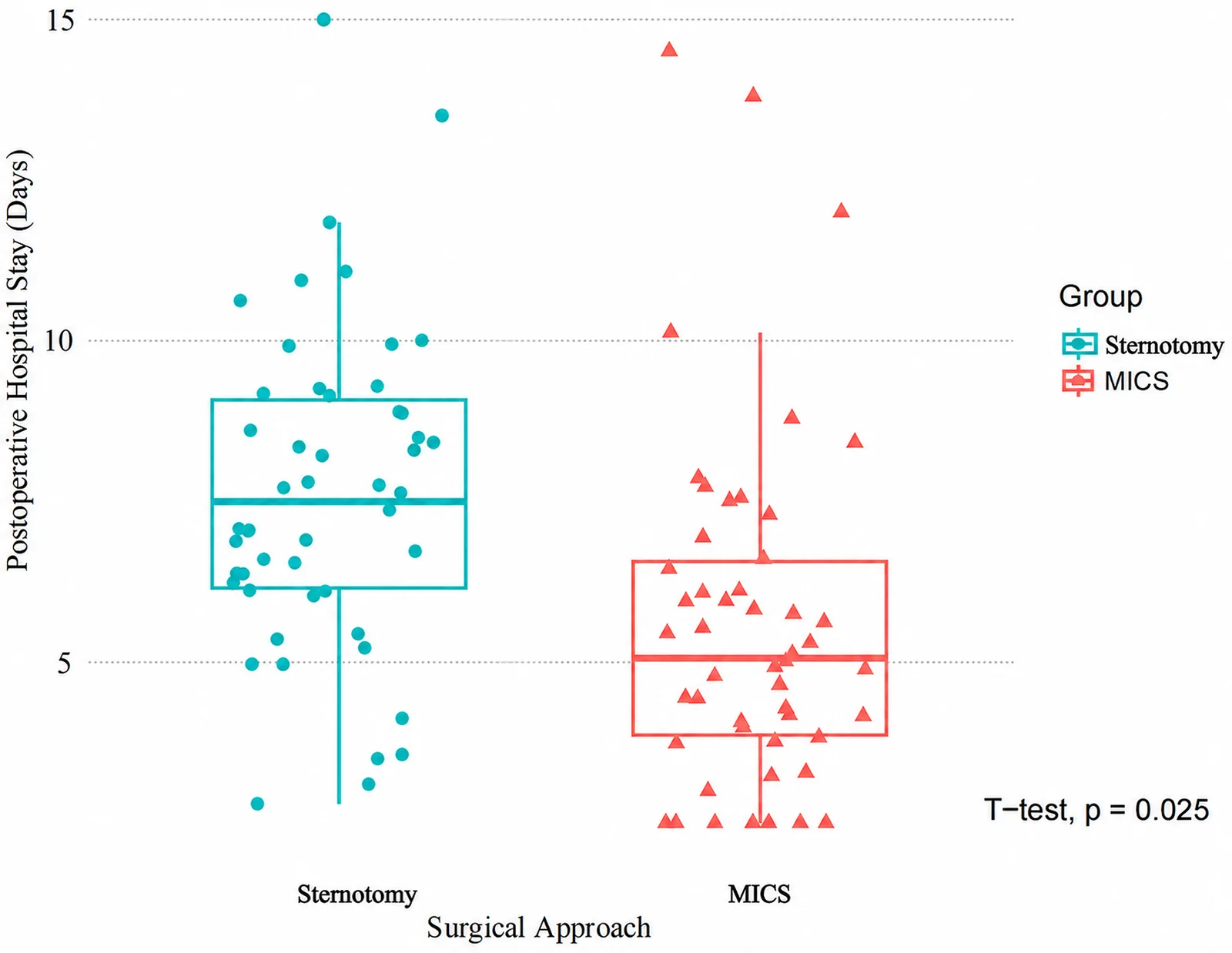
**Distribution of postoperative length of stay by surgical approach**. MICS in the figure label denotes the MICS-CABG group, defined as minimally invasive coronary artery bypass grafting via left mini-thoracotomy. The box plot shows the median, interquartile range, and individual data points. The median postoperative length of stay was lower in the MICS group than in the sternotomy group. The variability in length of stay was smaller in the MICS group, whereas the sternotomy group showed greater variability, including some longer stays. CABG, coronary artery bypass grafting; MICS-CABG, Minimally invasive CABG.

Covariate balance assessed by SMDs is shown in Table 2 and visualized in the Love plot (Fig. 2). Before matching, several baseline covariates showed meaningful imbalance, most notably prior PCI (SMD = 0.582), LVEF (SMD = 0.323), diabetes (SMD = 0.307), smoking status (SMD = 0.289), and male sex (SMD = 0.285). After matching, overall balance improved substantially, with most covariates achieving an SMD <0.10, including prior PCI (SMD = 0.082) and LVEF (SMD = 0.057). A modest residual imbalance remained for body mass index (SMD = 0.144) and eGFR (SMD = 0.169).

**Table 2.** **Covariate balance before and after propensity score matching (standardized mean differences)**.

Characteristic	Pre-PSM (Sternotomy N = 142)	Pre-PSM (MICS N = 66)	SMD	After-PSM (Sternotomy N = 53)	After-PSM (MICS N = 53)	SMD
Age, years	62.80 (8.72)	60.89 (9.81)	0.205	61.68 (10.18)	61.85 (9.76)	0.017
Sex, male, n (%)	99 (69.7)	54 (81.8)	0.285	42 (79.2)	42 (79.2)	0.001
BMI (kg/m^2^)	25.66 (3.09)	25.31 (3.19)	0.113	25.89 (3.17)	25.42 (3.30)	0.144
Hypertension (%)	74 (52.1)	33 (50.0)	0.042	27 (50.9)	27 (50.9)	0.001
Diabetes (%)	73 (51.4)	24 (36.4)	0.307	21 (39.6)	22 (41.5)	0.038
Smoking (%)	77 (54.2)	45 (68.2)	0.289	33 (62.3)	34 (64.2)	0.039
Hyperlipidemia (%)	43 (30.3)	15 (22.7)	0.172	12 (22.6)	12 (22.6)	0.001
History of stroke (%)	22 (15.5)	14 (21.2)	0.148	10 (18.9)	9 (17.0)	0.049
History of PCI (%)	24 (16.9)	28 (42.4)	0.582	17 (32.1)	15 (28.3)	0.082
eGFR (mL/min/1.73 m^2^)	87.26 (86.40)	91.07 (100.53)	0.041	78.29 (20.46)	91.90 (111.86)	0.169
LVEF (%)	54.71 (9.96)	57.77 (8.97)	0.323	56.49 (8.65)	57.01 (9.52)	0.057
EuroSCORE II	1.52 (0.88)	1.32 (0.57)	0.265	1.32 (0.54)	1.33 (0.51)	0.024
MR grade	0.90 (0.67)	0.73 (0.54)	0.287	0.79 (0.69)	0.75 (0.55)	0.060
AR grade	0.33 (0.47)	0.33 (0.48)	0.005	0.26 (0.45)	0.28 (0.45)	0.042
TR grade	0.38 (0.52)	0.41 (0.53)	0.055	0.40 (0.53)	0.38 (0.53)	0.036

Continuous variables are presented as mean (SD), and categorical variables as n (%). Standardized mean differences (SMDs) were used to assess covariate balance; an SMD <0.10 was considered indicative of adequate balance. Abbreviations: BMI, body mass index; MICS, label for the MICS-CABG group; eGFR, estimated glomerular filtration rate; LVEF, left ventricular ejection fraction; PCI, percutaneous coronary intervention; MR, mitral regurgitation; AR, aortic regurgitation; TR, tricuspid regurgitation; PSM, propensity score matching; EuroSCORE II, European System for Cardiac Operative Risk Evaluation II.

**Fig. 2.**
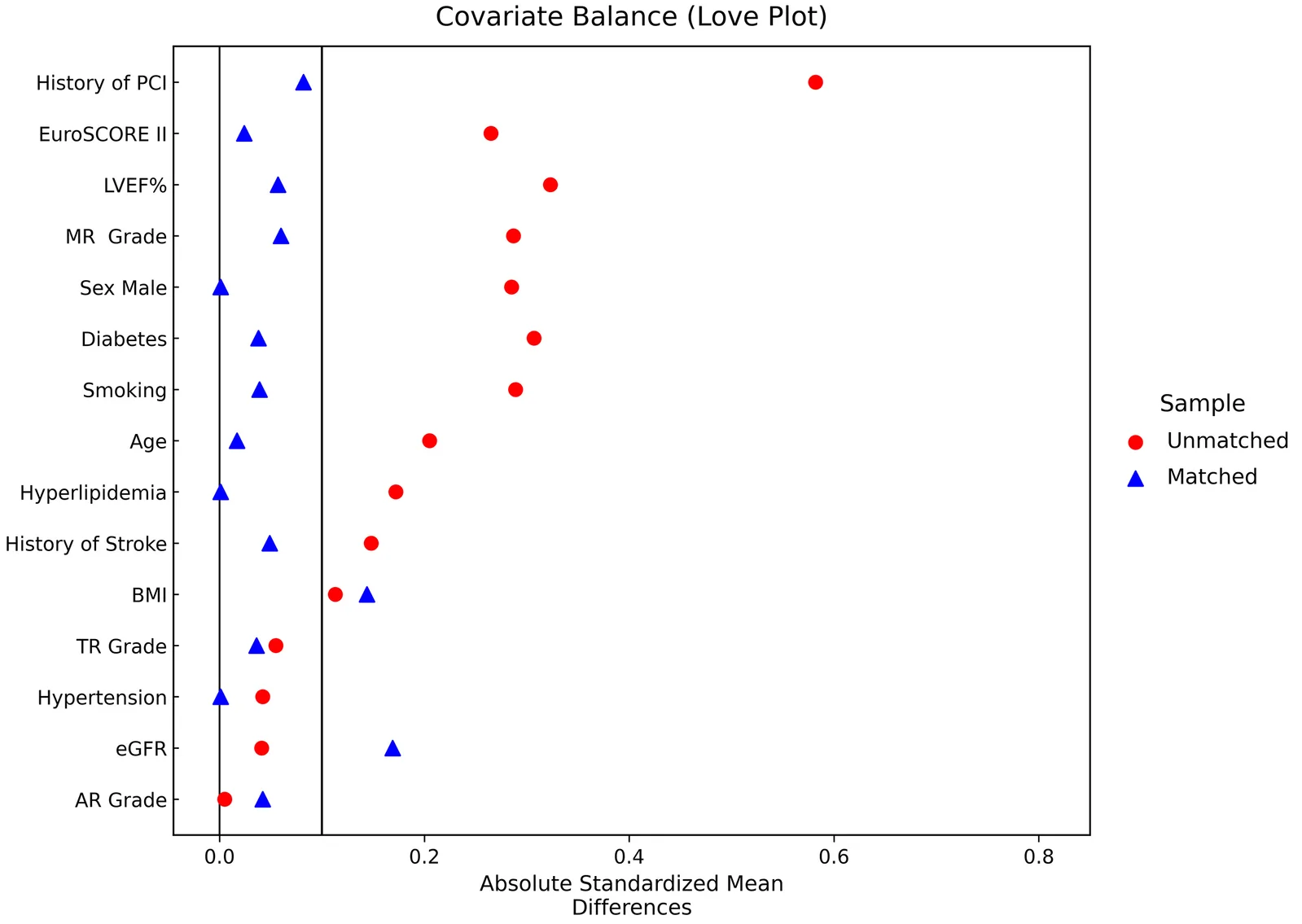
**Love plot of the covariate balance before and after propensity score matching**. The matching process improved covariate balance between the two groups across measured patient characteristics.

Linear regression analyses for postoperative LOS are summarized in Table 3. In univariable analysis, MICS-CABG was associated with shorter LOS (β = −1.14 days; 95% CI, −2.00 to −0.29; *p* = 0.009). Diabetes was associated with longer LOS (β = 0.82 days; 95% CI, 0.01 to 1.62; *p* = 0.046), whereas age, sex, BMI, EuroSCORE II, LVEF, CPB use, and number of grafts were not significantly associated with LOS (all *p* > 0.05). In the multivariable model including MICS-CABG, age, diabetes, EuroSCORE II, LVEF, CPB use, and number of grafts, MICS-CABG was no longer significantly associated with LOS (β = −0.67 days; 95% CI, −1.82 to 0.47; *p* = 0.247). Lower LVEF was independently associated with longer postoperative LOS (β = −0.08 per 1% increase; 95% CI, −0.15 to −0.02; *p* = 0.012). Age, diabetes, EuroSCORE II, CPB use, and number of grafts were not significantly associated with LOS after adjustment (all *p* > 0.05). In a sensitivity analysis excluding CPB use and number of grafts, the association between MICS-CABG and postoperative LOS remained directionally consistent but did not reach statistical significance (β = −0.83 days; 95% CI, −1.95 to 0.29; *p* = 0.145) (**Supplementary Table 1**).

**Table 3.** **Univariable and multivariable linear regression analyses for postoperative length of stay**.

Term	Univariable β (95% CI)	*p*	Multivariable β (95% CI)	*p*
MICS	−1.14 (−2.00, −0.29)	0.009	−0.67 (−1.82, 0.47)	0.247
Age	0.02 (−0.02, 0.07)	0.303	0.01 (−0.05, 0.07)	0.765
Sex, male	0.43 (−0.49, 1.34)	0.360	—	—
BMI	0.11 (−0.02, 0.24)	0.104	—	—
Diabetes	0.82 (0.01, 1.62)	0.046	0.22 (−0.93, 1.38)	0.706
EuroSCORE II	−0.05 (−0.56, 0.46)	0.852	−0.22 (−0.74, 0.30)	0.411
LVEF (%)	0.01 (−0.04, 0.05)	0.810	−0.08 (−0.15, −0.02)	0.012
CPB use	0.65 (−1.12, 2.42)	0.471	−0.47 (−3.93, 3.00)	0.790
Number of grafts	−0.19 (−0.64, 0.26)	0.405	0.43 (−0.23, 1.08)	0.198

Values are regression coefficients (β) with 95% confidence intervals (CIs). β represents the mean change in postoperative length of stay (days) per one-unit increase in the predictor (or presence vs. absence for binary variables). The multivariable model included MICS, age, diabetes, EuroSCORE II, LVEF, CPB use, and number of grafts. “—” indicates variables not included in the multivariable model. Abbreviations: CPB, cardiopulmonary bypass; LVEF, left ventricular ejection fraction; MICS, label for the MICS-CABG group.

Prespecified subgroup analyses of LOS are summarized in Fig. 3. Point estimates generally favored MICS-CABG across most strata, although CIs were wide. The overall mean difference was −0.97 days (95% CI, −2.24 to 0.30; *p* = 0.134). A larger reduction was observed among patients without hypertension (mean difference, −1.94 days; 95% CI, −3.60 to −0.28; *p* = 0.023), whereas the estimate was attenuated among patients with hypertension (0.16 days; 95% CI, −1.77 to 2.09; *p* = 0.869). Estimates in other subgroups were directionally consistent but not statistically significant.

**Fig. 3.**
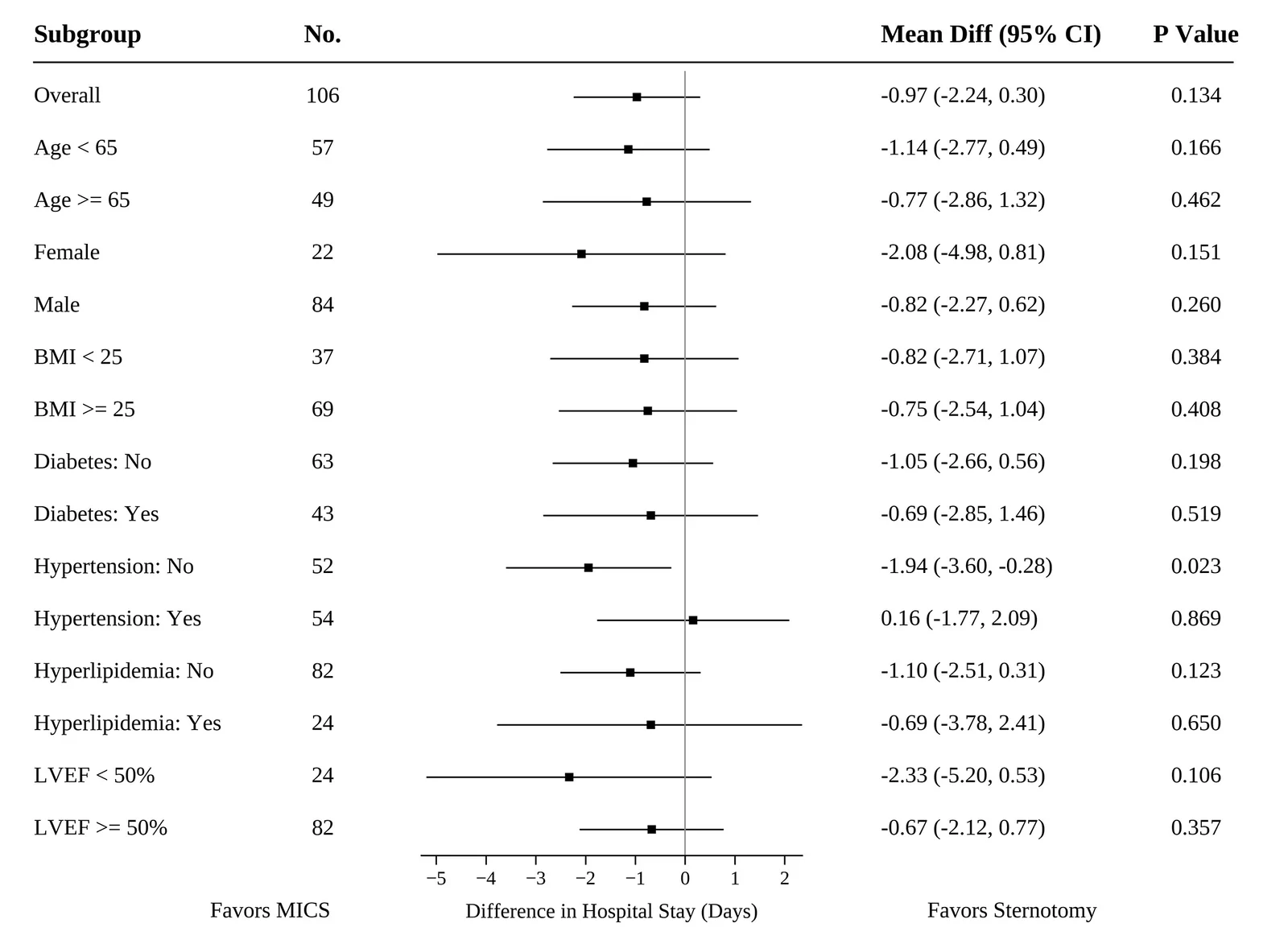
**Subgroup analysis of differences in hospital stay between MICS and sternotomy**. MICS in the figure label denotes the MICS-CABG group, defined as minimally invasive coronary artery bypass grafting via left mini-thoracotomy. MICS was associated with a shorter hospital stay only among patients without hypertension; no significant differences were observed in other subgroups or overall. Estimates represent stratum-specific mean differences. The subgroup analyses were exploratory and not powered to test for interactions.

Learning-curve analyses are shown in Fig. 4. Operative time plotted against case sequence demonstrated an early phase followed by relative stabilization. CUSUM analysis identified a peak at case 19, after which the CUSUM declined, consistent with a transition after the initial learning phase. Although the matched cohort included patients from both early and later phases of the learning curve, no increase in perioperative complications or adverse clinical outcomes was observed during the early phase.

**Fig. 4.**
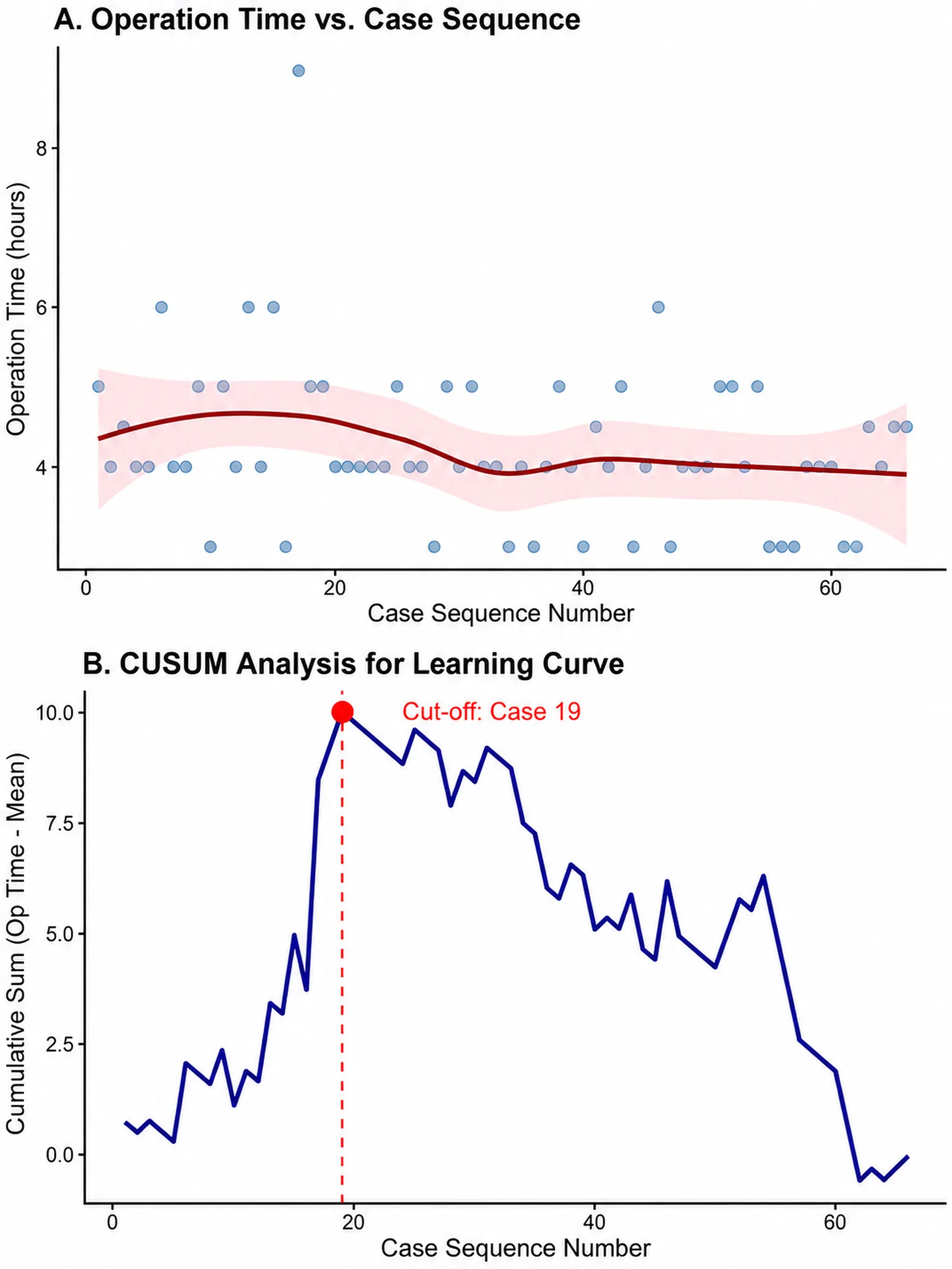
**Learning curve assessment**. (A) Operative time by case sequence. (B) Cumulative sum (CUSUM) analysis of the inflection point at around case 19.

## 4. Discussion

In this propensity score-matched retrospective study of patients with prior MI undergoing isolated CABG, the primary outcome was the occurrence of MACEs, reflecting the safety and adequacy of surgical myocardial revascularization. After matching, rates of in-hospital mortality, MACEs, and use of advanced circulatory or renal support were low and comparable between the minimally invasive left mini-thoracotomy approach and conventional sternotomy CABG. Adequate covariate balance after matching supports the validity of these comparisons. For recovery-related outcomes, the minimally invasive approach was associated with shorter postoperative LOS in both unmatched and matched analyses. However, this association was attenuated and no longer significant after multivariable adjustment, suggesting that LOS may also be influenced by patient- and procedure-related factors beyond surgical access alone.

Sensitivity analyses excluding procedural variables yielded consistent results, suggesting that potential mediation effects did not materially influence the overall findings. From a clinical perspective, the potential recovery benefit associated with minimally invasive access is biologically plausible. Avoidance of sternotomy may reduce musculoskeletal trauma, facilitate earlier mobilization, and improve postoperative respiratory mechanics, all of which may contribute to earlier hospital discharge [15,16,17,18]. In the present study, the distribution of LOS in the MICS-CABG group showed a consistent shift toward shorter hospital stays. Nevertheless, subgroup analyses showed wide CIs and a non-significant overall estimate, suggesting limited statistical power to detect subgroup-specific effects. Therefore, these exploratory findings require cautious interpretation and confirmation in larger cohorts.

A central concern raised by the present findings is the lower number of grafts in the MICS-CABG group, which may reflect differences in the extent of surgical revascularization. In patients with prior MI and complex CAD, the primary goal of CABG is not merely surgical access but the achievement of effective and durable myocardial revascularization, which has been strongly linked to improved long-term survival and reduced recurrent ischemic events in multiple studies [19,20,21,22,23,24]. Incomplete revascularization has been associated with worse long-term outcomes, particularly in patients with multivessel disease and impaired ventricular function. Thus, any strategy that potentially limits the completeness of revascularization must be carefully evaluated in the context of these established clinical priorities.

Revascularization relative to coronary disease burden was further evaluated using the diseased/transplanted vessel ratio. Although the absolute number of grafts was lower in the MICS-CABG group, categorical analyses of graft distribution showed no statistically significant difference in the matched cohort; notably, the diseased/transplanted vessel ratio was identical between groups (median 1.0 in both groups). However, this ratio should be interpreted as an exploratory and relatively crude metric rather than definitive evidence of comparable anatomical completeness of revascularization. These observed differences may reflect variation in coronary anatomy, target-vessel suitability, or conduit selection rather than systematic under-revascularization in the minimally invasive group. Nevertheless, detailed anatomical metrics such as SYNTAX score, target-vessel quality, and conduit strategy were not available in this dataset, and these factors may influence both surgical strategy and long-term outcomes. Notably, not all angiographically diseased vessels are amenable to grafting in clinical practice. Factors such as poor distal vessel quality, diffuse disease, or small vessel caliber may limit graftability. Therefore, the diseased/transplanted vessel ratio may overestimate the theoretical completeness of revascularization in some cases.

Patient selection remains an important consideration because the choice of MICS-CABG is influenced by anatomical and procedural factors that may not be fully captured in retrospective datasets.

The learning-curve analyses provide additional insight into the implementation of MICS-CABG. Operative time demonstrated an early phase of variability followed by stabilization, and the CUSUM analysis identified a transition around case 19. Although no increase in perioperative complications or adverse clinical outcomes was observed during the early phase, potential learning-curve effects cannot be entirely excluded. Therefore, the CUSUM findings should be interpreted as exploratory rather than as a prespecified basis for excluding patients from the matched cohort. Overall, including the full case sequence may better reflect real-world practice during implementation of the MICS-CABG program.

## 5. Limitations

This study has limitations inherent to single-center retrospective comparisons. Residual confounding may persist despite matching, including confounding related to anatomical complexity, frailty, pulmonary reserve, surgeon experience, case selection, pain and mobilization protocols, and institutional discharge practices. SYNTAX score, target-vessel quality, and detailed conduit strategy were not systematically recorded, limiting assessment of anatomical complexity and graftability. The extremely low event rate, with zero events in both matched groups, limited statistical power and precluded meaningful statistical comparison of the primary outcome. LOS may also be influenced by institutional pathways and resource availability, limiting the generalizability of this finding. Learning-curve effects identified by CUSUM may have influenced operative strategy and postoperative recovery; however, a sensitivity analysis excluding the early learning phase was not performed because of sample size constraints. Therefore, the CUSUM findings should be interpreted as exploratory rather than as a prespecified basis for excluding patients from the matched cohort. This study evaluated only in-hospital outcomes; thus, long-term endpoints, including survival, recurrent MI, repeat revascularization, and heart failure hospitalization, remain necessary to assess the durability of MICS-CABG in patients with prior MI. Finally, subgroup analyses involved multiple comparisons and should be interpreted with caution [24].

## 6. Conclusion

In summary, among patients with prior MI undergoing isolated CABG, MICS-CABG via left mini-thoracotomy showed an early in-hospital safety profile similar to that of sternotomy CABG after PSM. Shorter postoperative LOS was observed in unadjusted and matched analyses, but this association was attenuated and was not independently associated with surgical approach after multivariable adjustment. Larger multicenter studies incorporating anatomical complexity and longer-term follow-up are needed to clarify generalizability, durability, and the impact of completeness of revascularization.

## Data Availability

The data presented in this study are available on request from the corresponding author.

## Abbreviations

MI, myocardial infarction; CABG, coronary artery bypass grafting; CI, confidence interval; MICS-CABG, minimally invasive coronary artery bypass grafting via left mini-thoracotomy; LOS, length of stay; PSM, propensity score matching; MACEs, major adverse cardiovascular events; CPB, cardiopulmonary bypass; CAD, coronary artery disease; PCI, percutaneous coronary intervention; MIDCAB, minimally invasive direct coronary artery bypass grafting; LVEF, left ventricular ejection fraction; ICU, intensive care unit; IABP, intra-aortic balloon pump; ECMO, extracorporeal membrane oxygenation; CRRT, continuous renal replacement therapy; SMDs, standardized mean differences; CUSUM, cumulative sum.
