# Supplementary material for: Minimally Invasive Coronary Artery Bypass Grafting in Patients With Prior Myocardial Infarction: A Propensity Score-Matched Retrospective Study
Source: Rev Cardiovasc Med. 2026 Sep 16;27(9):49786. doi: 10.31083/RCM49786 (PMC13653189; doi:10.31083/RCM49786)
Supplement: Supplementary file 1 [file 2153-8174-27-9-49786-s1.zip › Supplementary Table 1.docx]

Supplementary Table 1. Univariable, multivariable, and sensitivity analyses using linear regression models for postoperative length of stay.

| **Term** | **Univariable β (95% CI)** | ***p*** | **Original Multivariable β (95% CI)** | ***p*** | **Sensitivity Multivariable β (95% CI)** | ***p*** |
| --- | --- | --- | --- | --- | --- | --- |
| MICS | −1.14 (−2.00, −0.29) | 0.009 | −0.67 (−1.82, 0.47) | 0.247 | −0.83 (−1.95, 0.29) | 0.145 |
| Age | 0.02 (−0.02, 0.07) | 0.303 | 0.01 (−0.05, 0.07) | 0.765 | 0.00 (−0.06, 0.06) | 0.936 |
| Sex, male | 0.43 (−0.49, 1.34) | 0.360 | — | — | — | — |
| BMI | 0.11 (−0.02, 0.24) | 0.104 | — | — | — | — |
| Diabetes | 0.82 (0.01, 1.62) | 0.046 | 0.22 (−0.93, 1.38) | 0.706 | 0.24 (−0.92, 1.39) | 0.685 |
| EuroSCORE II | −0.05 (−0.56, 0.46) | 0.852 | −0.22 (−0.74, 0.30) | 0.411 | −0.16 (−1.28, 0.96) | 0.779 |
| LVEF (%) | 0.01 (−0.04, 0.05) | 0.810 | −0.08 (−0.15, −0.02) | 0.012 | −0.08 (−0.15, −0.02) | 0.013 |
| CPB use | 0.65 (−1.12, 2.42) | 0.471 | −0.47 (−3.93, 3.00) | 0.790 | — | — |
| Number of grafts | −0.19 (−0.64, 0.26) | 0.405 | 0.43 (−0.23, 1.08) | 0.198 | — | — |
| Values are presented as regression coefficients (β) with 95% confidence intervals. β indicates the mean change in postoperative length of stay (days) associated with a one-unit increase in the predictor (or the presence vs. absence of a binary variable). The multivariable model included MICS, age, diabetes, EuroSCORE II, LVEF, CPB use, and number of grafts. “—” indicates variables that were not included in the multivariable model.  Abbreviations: CPB, cardiopulmonary bypass; LVEF, left ventricular ejection fraction; MICS, label for the MICS-CABG group. | | | | | | |
